# Podemos Realizar o Teste de Esforço Máximo em Esteira em Indivíduos com Doença Falciforme?

**DOI:** 10.36660/abc.20220036

**Published:** 2022-03-10

**Authors:** Mabel Marciela Ahner, Stephanie Bastos da Motta, Leandro Franzoni

**Affiliations:** 1 Pontifícia Universidade Católica do Rio Grande do Sul Porto Alegre RS Brasil Pontifícia Universidade Católica do Rio Grande do Sul, Porto Alegre, RS - Brasil; 2 Hospital de Clínicas de Porto Alegre Grupo de Vascular e Exercício VascuEx Porto Alegre Brasil Hospital de Clínicas de Porto Alegre - Grupo de Vascular e Exercício – VascuEx, Porto Alegre, RS – Brasil

**Keywords:** Anemia Falciforme, Teste de esforço

A doença falciforme (DF) pode apresentar quadro clínico estável com o avanço do tratamento farmacológico e das tecnologias disponíveis para o diagnóstico precoce.^1^ No entanto, se não diagnosticada e tratada precocemente, pode levar a lesões progressivas de órgãos e até complicações fatais.^2^ Portanto, é importante desenvolver diferentes instrumentos para avaliar o prognóstico da DF. O teste de esforço máximo (TEM), amplamente utilizado em diversas doenças, como a insuficiência cardíaca, pode desempenhar um papel importante na estratificação de risco desses pacientes, que geralmente apresentam dor torácica associada à oclusão do vaso, causando isquemia miocárdica e, consequentemente, morte súbita, algo muito comum nesses indivíduos.^3 , 4^

No entanto, pacientes com DF precisam ter cautela ao realizar exercícios físicos, principalmente de alta intensidade, pois esses exercícios podem levar a distúrbios metabólicos que podem favorecer a falcização eritrocitária e promover oclusões vasculares.^5^ Esse fato levantou um debate e um dilema entre recomendar exercícios físicos para esses pacientes ou privá-los dos efeitos positivos que o exercício físico é capaz de promover.^6 , 7^ Devido à associação descrita acima, entre exercício físico e isquemia em indivíduos com DF, é necessário realizar um teste de esforço.^8^ No entanto, chegamos ao paradoxo do risco versus benefício. Indivíduos com DF podem realizar TEM com segurança para fornecer respostas sobre o impacto cardiovascular induzido pelo esforço na ocorrência de desfechos clínicos? Foi o que fizeram Araújo et al.,^9^ A seguir descreveremos as principais características do estudo e seus principais resultados.

Este é um estudo observacional que teve como objetivo avaliar a segurança e viabilidade de um TEM em pacientes com DF. Além disso, foram avaliados fatores associados à duração do teste e o impacto das alterações causadas pelo teste nos desfechos clínicos. Para o desenvolvimento do estudo, foram incluídos 133 pacientes com DF. Além de serem submetidos a uma avaliação de esforço físico, eles foram submetidos a uma avaliação cardiovascular abrangente, incluindo ecocardiograma, bem como os níveis de peptídeo natriurético tipo B (BNP). O desfecho de longo prazo (24 meses) foi uma combinação de eventos, como mortalidade, crises de dor intensa (crises álgicas), síndrome torácica aguda ou internações hospitalares por outras complicações associadas à doença.

Devemos chamar a atenção para os resultados encontrados, como alterações isquêmicas ao esforço, detectadas em 17% (19) dos pacientes, e também alterações na pressão arterial (PA) durante o teste, detectadas em 9% (10). Esses dados já nos trazem um alerta para a avaliação ergométrica nessa população. Em relação às respostas agudas mais graves, como crises de dor, 48 horas após o exame, dois pacientes necessitaram de internação. Os fatores associados à duração do teste são idade, sexo, pico de velocidade de regurgitação tricúspide (VRT) e razão E/e’, todos marcadores padronizados de gravidade da doença. 23% dos pacientes tiveram algum desfecho clínico adverso, com seguimento médio de 10,1 meses (variando de 1,2 a 26). Preditores independentes de eventos adversos foram concentração de hemoglobina, velocidade de fluxo transmitral tardia (onda A) e resposta da PA ao esforço físico.

Citaremos algumas limitações do presente estudo a fim de melhorar a condução de estudos futuros, pois o tema é muito interessante e carece de literatura científica robusta. Uma das limitações é que o tamanho da amostra foi estimado de modo a detectar alterações eletrocardiográficas relacionadas à isquemia miocárdica em indivíduos com DF, porém, sem levar em consideração a análise de preditores de eventos adversos. Quando se trata de estudos científicos, devemos atentar para a validade interna e externa, que determina o poder de extrapolar os dados para uma amostra maior.^10^ Este estudo foi muito bem conduzido. No entanto, não possui boa validade externa, pois os pacientes foram encaminhados de um ambulatório com DF, mas com pequeno número de subgrupos mais graves, principalmente aqueles com hipertensão pulmonar, limitando a validade externa aos pacientes com quadros mais graves. A sugestão é realizar um ensaio clínico randomizado no futuro com subgrupos de diferentes níveis de gravidade da doença para melhor validação externa e consequentemente melhorar a qualidade das evidências.^11^

O que pode ser destacado positivamente é que o TEM para pacientes com DF é relativamente seguro e viável, oferecendo informações clínicas valiosas, além de ser útil na avaliação da condição aeróbia. Além disso, é possível concluir que a duração do teste está associada à função diastólica e à pressão arterial pulmonar e que uma resposta anormal da PA foi preditor independente de eventos adversos. Essas informações são de grande valia na realização de TEM em pacientes com DF.
