# Supplementary material for: Podemos Realizar o Teste de Esforço Máximo em Esteira em Indivíduos com Doença Falciforme?
Source: Arq Bras Cardiol. 2022 Mar 10;118(3):576–7. [Article in Portuguese] doi: 10.36660/abc.20220036 (PMC8959026; doi:10.36660/abc.20220036)
Supplement: Translation

# Translation: Can We Perform the Maximal Treadmill Test on Individuals with Sickle Cell Disease?

**Authors:** Mabel Marciela Ahner, Stephanie Bastos da Motta, Leandro Franzoni

**Affiliations:** 1 Brazil Pontifícia Universidade Católica do Rio Grande do Sul, Porto Alegre, RS - Brazil; 2 Brazil Hospital de Clínicas de Porto Alegre - Grupo de Vascular e Exercício – VascuEx, Porto Alegre, RS – Brazil

**Keywords:** Sickle Cell Anemias, Exercise Test

Sickle cell disease (SCD) may present a stable clinical condition with the advancement of pharmacological treatment and available technologies for early diagnosis.^1^ However, if not diagnosed and treated early, it may lead to progressive organ damage and even fatal complications.^2^ Therefore, it is important to develop different instruments to assess the SCD prognosis. The maximal treadmill test (MTT), widely used in different diseases, such as heart failure, can play an important role in the risk stratification of these patients, since they usually have chest pain associated with vessel occlusion, causing myocardial ischemia and, consequently, sudden death, something very common in these individuals.^3 , 4^

However, patients with SCD need to exercise caution when performing physical exercises, especially at high-intensity, as these may lead to metabolic disorders that could favor erythrocyte sickling and promote vascular occlusions.^5^ This fact raised a discussion and a dilemma between recommending physical exercise for these patients or depriving them of the positive effects that physical exercise is capable of promoting.^6 , 7^ Due to the association described above, between physical exercising and ischemia in SCD individuals, it is necessary to perform an exercise test.^8^ However, we get to the paradox of risk versus benefit. Can individuals with SCD safely perform a MTT to provide answers about the cardiovascular impact induced by exertion in the occurrence of clinical outcomes? This is what Araújo et al.,^9^ Below we will describe the main study characteristics and its main results.

This is an observational study that aimed to assess the safety and feasibility of a MMT in SCD patients. In addition, factors associated with test duration and the impact of changes caused by the test on clinical outcomes were evaluated. For the development of the study, 133 patients with SCD were included. In addition to undergoing an exercise stress assessment, they underwent a comprehensive cardiovascular assessment, including echocardiography, as well as B-type natriuretic peptide (BNP) levels. The long-term outcome (24 months) was a combination of events, such as mortality, severe pain crises, acute chest syndrome, or hospital admissions for other complications associated with the disease.

We need to draw attention to the results found, such as ischemic changes on exertion, which were detected in 17% (19) of the patients, and also to abnormal blood pressure (BP) responses during the test, detected in 9% (10). These data already bring us an alert to the ergometric evaluation in this population. Regarding more severe acute responses, such as pain crises, 48 hours after the test, two patients required hospitalization. The factors associated with the test duration include age, sex, maximum tricuspid regurgitation velocity (TRV) and E/e’ ratio, all standardized markers of disease severity. 23% of the patients had some adverse clinical outcome, with a mean follow-up period of 10.1 months (ranging from 1.2 to 26). Independent predictors of adverse events were hemoglobin concentration, late transmitral flow velocity (A wave), and BP response to physical exertion.

We will cite some limitations of the present study, in order to improve the conduct of future studies, as the topic is very interesting and lacks robust scientific literature. One of the limitations is that the sample size was estimated to detect electrocardiographic abnormalities related to myocardial ischemia in SCD individuals, however, without taking into account the analysis of predictors of adverse events. When it comes to scientific studies, we must pay attention to internal and external validity, which determines the power to extrapolate the data to a larger sample.^10^ This study was very well conducted. However, it does not have good external validity, as patients were referred from an outpatient clinic with SCD, but with a small number of more severe subgroups, especially those with pulmonary hypertension, limiting external validity to patients with more severe conditions. The suggestion is to conduct a randomized clinical trial in the future with subgroups of different levels of disease severity for better external validation and consequently improve the quality of evidence.^11^

What can be positively highlighted is that the MMT for SCD patients is relatively safe and feasible, offering valuable clinical information, in addition to being useful in the assessment of aerobic condition. Furthermore, it is possible to conclude that test duration is associated with diastolic function and pulmonary artery pressure and that an abnormal BP response was an independent predictor of adverse events. This information is supportive when performing a MMT in SCD patients.
